# Textile Bandwidth-Enhanced Half-Mode Substrate-Integrated Cavity Antenna Based on Embroidered Shorting Vias

**DOI:** 10.3390/mi15091081

**Published:** 2024-08-27

**Authors:** Feng-Xue Liu, Fan-Yu Meng, Yu-Jia Chen, Zhou-Hao Gao, Jie Cui, Le Zhang

**Affiliations:** 1 School of Physics and Electronic Engineering, Jiangsu Normal University, Xuzhou 221116, China; liufengxue@jsnu.edu.cn (F.-X.L.); 3020214557@jsnu.edu.cn (F.-Y.M.); zhangle@jsnu.edu.cn (L.Z.); 2 Jiangsu Xiyi Advanced Materials Research Institute of Industrial Technology, Xuzhou 221400, China; 3 JSNU SPBPU Institute of Engineering, Jiangsu Normal University, Xuzhou 221116, China; chenyujia@jsnu.edu.cn (Y.-J.C.); gaozhouhao@jsnu.edu.cn (Z.-H.G.); 4 School of Transportation Engineering, Jiangsu Vocational Institute of Architectural Technology, Xuzhou 221116, China

**Keywords:** textile antenna, wearable antenna, half-mode substrate-integrated cavity antenna, bandwidth enhancement, embroidered shorting via

## Abstract

A textile bandwidth-enhanced half-mode substrate-integrated cavity (HMSIC) antenna based on embroidered shorting vias is designed. Based on the simulated results of the basic HMSIC antenna, two embroidered hollow posts with square cross-sections are added as shorting vias at the intersections of the zero-E traces of the ${TM}_{210}^{HM}$ and ${TM}_{020}^{HM}$ modes to shift the ${TM}_{010}^{HM}$-mode band to merge with the bands of the higher-order modes for bandwidth enhancement. A prototype is practically fabricated based on computerized embroidery techniques. Measurement results show that the prototype is of an expanded −10 dB impedance band of 4.87~6.17 GHz (23.5% fractional bandwidth), which fully covers the 5 GHz wireless local area network (WLAN) band. The simulated radiation efficiency and maximum gain of the proposed antenna are above 97% and 7.6 dBi, respectively. Furthermore, simulations and measurements prove its robust frequency response characteristic in the proximity of the human tissues or in bending conditions, and the simulations of the specific absorption rate (SAR) prove its electromagnetic safety on the human body.

## 1. Introduction

The wearable antenna has been widely studied and applied in multiple areas including healthcare [1,2,3], athletic sports [4,5,6], defense [7,8,9], and special operations [10,11,12]. The HMSIC or half-mode substrate-integrated waveguide (HMSIW) antenna acts as half a rectangular/circular resonant cavity, and is of the same resonance frequencies and internal electrical field distributions as the full-mode cavity [13,14]. The textile HMSIC antenna has the advantages of planar structure, small size and high flexibility/integratability on clothes, and is therefore a suitable candidate for wearable applications [15,16]. In a textile HMSIC antenna, the horizontal top layer and ground plane are usually formed by copper tapes, conductive fabrics or densely embroidered conductive threads, and vertical sidewalls defining the 2-D footprint of the half-cavity can be formed by metal rivets or linearly embroidered conductive threads. However, its highly frequency-selective cavity geometry usually leads to a limited bandwidth. Bandwidth enhancing allows the textile HMSIC antenna to yield a stable performance against the frequency shifting caused by the human body or physical deformations, and is conductive to reusing a single antenna in multiple frequency bands.

For the wearable/flexible HMSIC antenna, slots can be loaded on its top layer to shift its higher-order band or introduce an extra band to achieve bandwidth enhancement. Reference [17] introduced a flexible slotted HMSIC antenna with a widened −10 dB impedance band (23.7% fractional bandwidth). In reference [18], the bandwidth of the 2.45 GHz band of a textile dual-band HMSIC antenna increased from 4.5% to 6.0% by adding a slot. Our previous works [19,20] also showed that adding a V-shape/straight slot on the textile HMSIC antennas successfully led to a bandwidth enhancement at 5.5/2.42 GHz. However, the slot needs to be precisely cut on the flexible top layer of the antenna. And physical deformations of the flexible antenna in practical wearable applications can potentially change the slot width, and lead to a deterioration in the antenna’s performance.

On the other hand, adding shorting vias can also enhance the bandwidth for rigid HMSIC and patch antennas. In reference [21], shorting posts were added in a rigid patch antenna to enhance its −10 dB fractional bandwidth to 50.46%. Reference [22] also introduced a microstrip dipole antenna loaded by vias with an expanded −10 dB fractional bandwidth of 9.1%. Such a bandwidth-enhancing method has been proved in the literature to be effective for the wearable/flexible HMSIC antennas and planar inverted-F antennas (PIFAs). In our previous work [23], copper rivets and conductive embroidered knots were added to textile HMSIC antennas as shoring vias, and the band of the higher-order mode was shifted towards the counterpart of the fundamental mode to obtain enhanced −10 dB fractional bandwidths of 19.7% and 14.7%, respectively, at 5.5 GHz. In reference [24], shorting vias and slots were simultaneously added in an all-textile PIFA to shift the bands of the lower- and higher-order modes towards each other, and a 18% −10 dB fractional bandwidth was obtained at 5.5 GHz. It has been validated that adding shorting vias can shift the band of the lower-order mode towards that of the higher-order mode, but requires a disadvantageous larger cavity/patch rather than a slot-based solution. However, this method requires no precise cutting on the top layer and yields a higher durability against physical deformations.

This article presents a simple and effective via-based bandwidth-enhancing method for the textile HMSIC antenna. Firstly, a basic antenna is designed based on the semicircular HMSIC, and the corresponding fundamental and higher-order modes are recognized based on the simulation results of the electrical field. Secondly, simulations are carried out to investigate the influence of adding two square shorting vias at the intersections of the zero-E traces of the two higher-order modes, and a textile bandwidth-enhanced HMSIC antenna is therefore designed for 5 GHz WLAN applications. The antenna performances in terms of frequency response, radiation efficiency, and radiation patterns in free space and on the phantom model are analyzed through simulations. Lastly, a prototype of the proposed textile antenna is fabricated, and its return loss coefficient and radiation patterns are measured for verification.

## 2. Analysis of Basic HMSIC Antenna and Its Internal Electrical Fields

**Figure 1:**
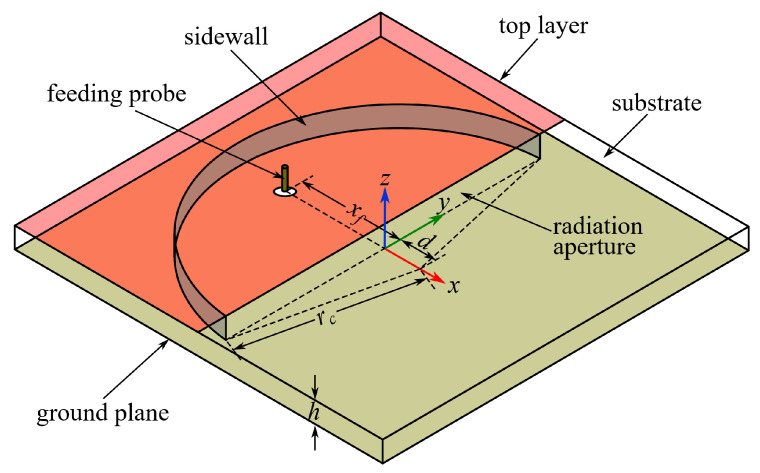
Geometries of basic HMSIC antenna (Ant I).

A basic HMSIC antenna (denoted as Ant I) is shown in Figure 1. Although Ant I will be practically fabricated, it is designed in this section with the same materials as the textile geometry in Section 3 for comparison. The substrate layer is made of PF-4 foam (relative permittivity *ε_r_* = 1.06 and dielectric loss tangent tan *δ* = 0.0001) with a thickness *h* of 3.2 mm. Its ultra-low loss tangent enables the antenna to exhibit an advantageous and desired high efficiency, and its excellent resilience against physical deformations guarantees consistent antenna performances before/after bending tests. Both the top layer and ground plane are made of the copper/nickel-coated polyester fabric (surface resistance *r_s_* = 0.04 Ω/sq from data sheet). The ground plane is designed to be a 110 × 110 mm^2^ square to provide sufficient shielding for the human body from antenna radiation, and the top layer yields half the 2-D footprint of the ground plane to allow the radiation aperture across the center of the geometry. The sidewall is formed by conductive threads linearly embroidered for three passes with a stitching space of 1.5 mm, and is modelled as a cylindrical surface (sheet resistance *r_s_* = 0.6 Ω/sq according to preliminary measurements). The feeding probe is modelled as a solid copper post with a 1.2 mm diameter to imitate the central pin of the SMA connector.

A semicircular cavity is selected for Ant I for its larger bandwidth compared to the rectangular counterpart [15]. The resonance frequency of the ${TM}_{mnl}^{HM}$ mode can be theoretically calculated by [25]:

(1) $$f_{r}=\frac{K_{mnl}c}{2\pi r_{c}\sqrt{\varepsilon_{r}}}$$

where *c* and *r_c_* represent the free-space light velocity and the cavity radius, respectively, and *K*_mnl_ is the Bessel’s coefficient (*K*_010_ = 2.4048, *K*_210_ = 5.135, *K*_020_ = 5.520).

Based on (1) and Hfss parameter optimizations, *r_c_* is determined to be 47 mm. The center of the cylindrical sidewall is offset from the radiation aperture by *d* = 4 mm to offset the influence of the fringing field [26], and the optimized feeding position *x_f_* = 29.5 mm guarantees the impedance matching at each resonance frequency. The values of parameters *d* and *x_f_* are also optimized through simulations in Hfss.

**Figure 2:**
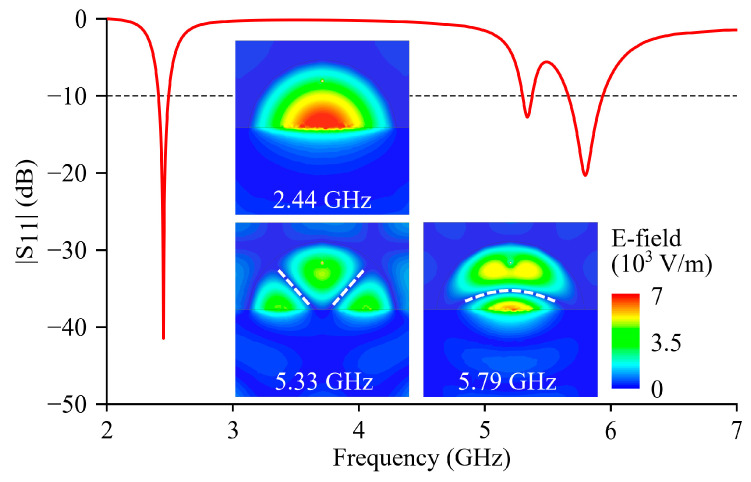
Simulated |S_11_| curve of Ant I.

For Ant I, the return loss coefficient (|S_11_|) is simulated from 2 GHz to 7 GHz, and the simulated curve is shown in Figure 2. Three resonance frequencies *f_r_*_1_, *f_r_*_2_, and *f_r_*_3_ are observed from the simulated |S_11_| curve at 2.44, 5.33, and 5.79 GHz, respectively. The simulated −10 dB fractional bandwidths of Ant I at three resonance frequencies are 3.4%, 1.6%, and 5%, respectively. The relatively low bandwidths of Ant I mainly result from the use of low-loss materials [27], but the low losses also lead to a high radiation efficiency (≥95% in simulations).

The internal electrical field distributions are simulated and shown in the insets of Figure 2, and the ${TM}_{010}^{HM}$, ${TM}_{210}^{HM}$ and ${TM}_{010}^{HM}$ modes can be recognized at 2.44, 5.33, and 5.79 GHz, respectively [18]. For the ${TM}_{210}^{HM}$ and ${TM}_{010}^{HM}$ modes, the traces where the electrical field is close to zero (denoted as zero-E traces) are marked by white dashed lines. Through measurements on the marked traces, the zero-E traces of the two higher-order modes approximately intersect at (*x_v_*, ±*y_v_*) where *x_v_* = 8.5 mm and *y_v_* = 16 mm.

## 3. Geometry Design

**Figure 3:**
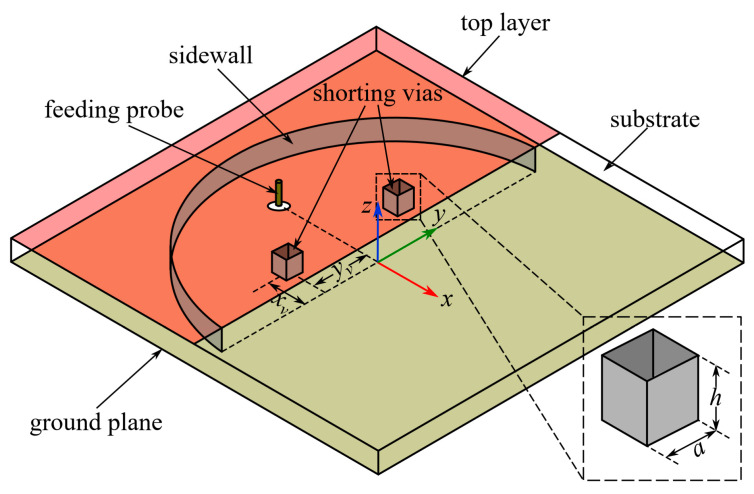
Geometries of textile bandwidth-enhanced HMSIC antenna based on embroidered shorting vias (Ant II).

Based on the analysis of the internal electrical field distributions, a textile bandwidth-enhanced HMSIC antenna (denoted as Ant II) as shown in Figure 3 is designed by adding two shorting vias. At the intersections of the zero-E traces of two higher-order modes, the electrical field is not zero for the original ${TM}_{010}^{HM}$ mode. Therefore, it is assumed that adding two shorting vias at these intersections leads to the shift of the band of the ${TM}_{010}^{HM}$ mode towards a higher frequency without changing *f_r_*_2_ and *f_r_*_3_. Considering *f_r_*_2_ and *f_r_*_3_ are already close to each other, a widened band can be obtained when the shift of the ${TM}_{010}^{HM}$-mode band is large enough to allow the merging of all three bands.

Ant II is of the same materials and values of dimensional parameters (except *x_f_*) as Ant I in Section 2. The added shorting vias at the intersections of the zero-E traces are modelled as conductive hollow posts, and will be formed by the same linear embroidery of conductive threads performed for the sidewall. The cross-section of the shorting via is determined to be square for the sake of easy embroidery fabrication with a side length *a*. Compared to the metallic rivets, the embroidered shorting vias can be built by the same threads, embroidery machine, and fabrication procedures that would be used for the sidewall, and are modeled with the same equivalent sheet resistance as the sidewall.

**Figure 4:**
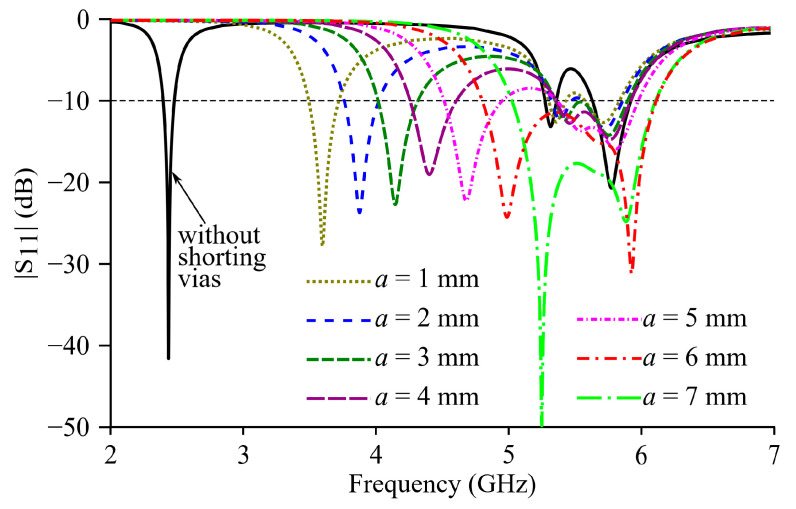
Simulated |S_11_| curves of Ant II with different values of *a*.

Parametric studies are carried out for Ant II through simulations. As shown in the simulated |S_11_| curves in Figure 4, *f_r_*_1_ rises when *a* increases, and the slight increases in *f_r_*_2_ and *f_r_*_3_ can be ignored compared to that of *f_r_*_1_. Parameter *a* is eventually determined to be 6 mm to allow the bands of all three modes to be merged to obtain a maximum bandwidth. Furthermore, parameter *x_f_* is re-optimized to be 24 mm through simulations for Ant II to achieve impedance matching.

**Figure 5:**
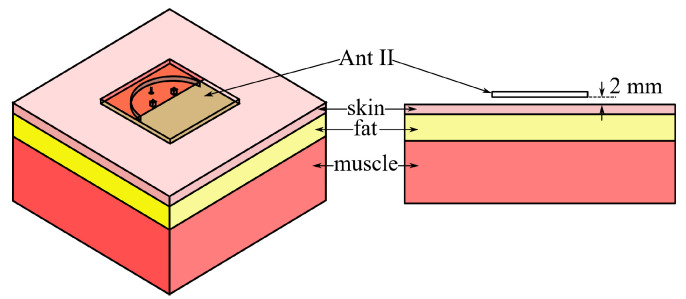
Geometry of the three-layer phantom model.

The performance of Ant II on the human body is investigated through simulations by using a 300 × 300 × 60 mm^3^ phantom model as shown in Figure 5. This phantom model consists of three layers of skin, fat, and muscle, and the employed relative permittivities, conductivities at 5.5 GHz and densities, as well as thicknesses of each layers in modelling are listed in Table 1 [24]. In simulations, the phantom model is located below the ground plane of Ant II with its center axis vertically coinciding with that of the antenna, and the distance between Ant II and the phantom model is selected to be 2 mm to imitate the practical wearable application where the antenna is attached to clothes worn on the human body.

## 4. Simulation Results and Analysis

**Figure 6:**
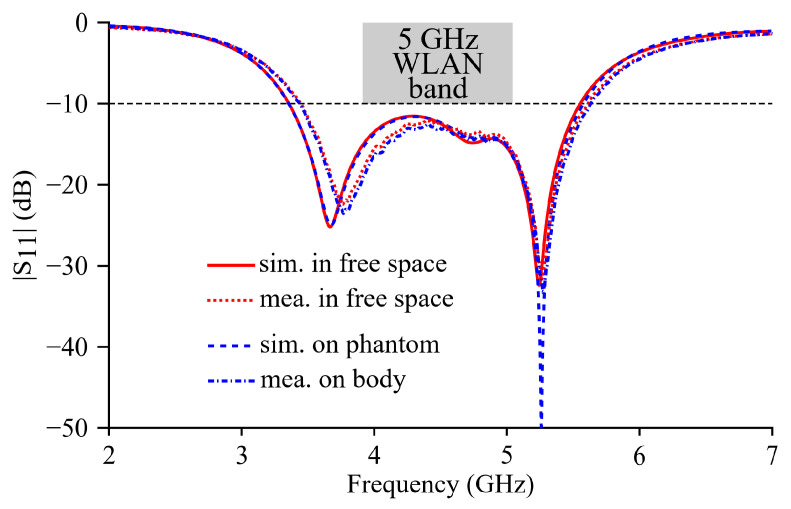
Simulated and measured return loss (|S_11_|) curves of Ant II in flat condition.

The return loss coefficients of Ant II in free space and on the phantom model are respectively simulated, and the simulated curves are shown in Figure 6. In free space, the simulated return loss is below −10 dB in 4.82~6.13 GHz (24% fraction bandwidth), which well covers the 5 GHz WLAN band (5.15~5.825 GHz), and the simulated *f_r_*_1_, *f_r_*_2_, and *f_r_*_3_ are 5, 5.65, and 5.95 GHz, respectively. It can be noticed from the simulated free-space curve that the notch at 5.65 GHz is not as deep as those at 5 and 5.95 GHz, but the corresponding return loss is still below −15 dB. On the phantom model, the simulated |S_11_| curve basically overlaps with the free-space counterpart with the same −10 dB impedance band and resonance frequencies. The simulation results indicate the robust frequency response characteristic of the proposed textile antenna against the influence of the human tissues.

**Figure 7:**
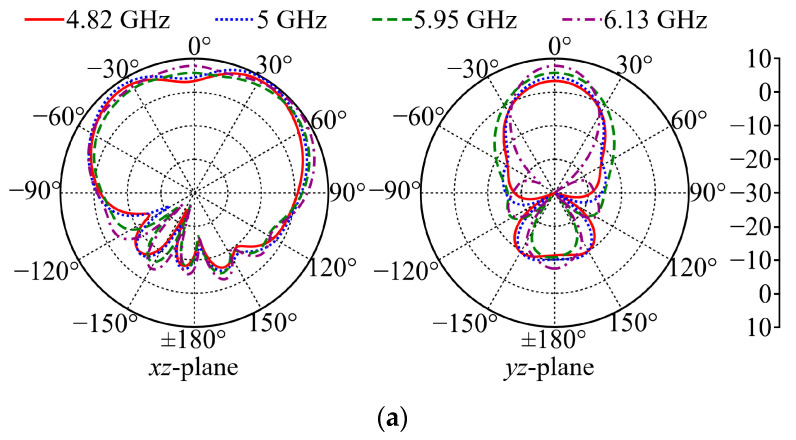

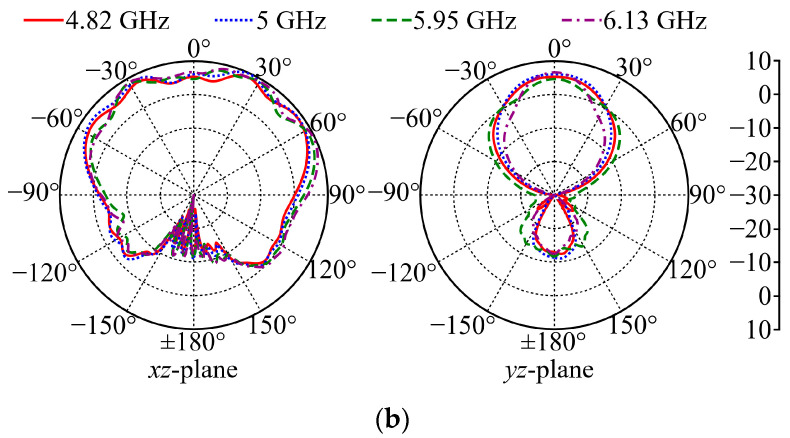
Simulated patterns of coplanar-polarized gain of Ant II in flat condition: (**a**) in free space; (**b**) on the phantom model.

The patterns of the coplanar-polarized gain of Ant II in free space at 4.82, 5, 5.95, and 6.13 GHz are simulated as shown in Figure 7a. At each frequency, the simulated maximum coplanar-polarized gains are 8.9, 9.7, 8.1, and 9.6 dBi, respectively. Figure 7b shows the simulated patterns of the coplanar-polarized gain of Ant II with the phantom model at 4.82, 5, 5.95, and 6.13 GHz. At each frequency, the simulated maximum co-polarized gains are 8.7, 9.8, 9.1, and 9.6 dBi, respectively. For both scenarios, the simulated patterns are of wide main beams in the positive semisphere and low backward radiations (≤−7 dB in free space and ≤−10 dB on the phantom model). The observed lower backward radiation of Ant II on the phantom model indicates that the phantom model partially reflects the backward radiation.

**Figure 8:**
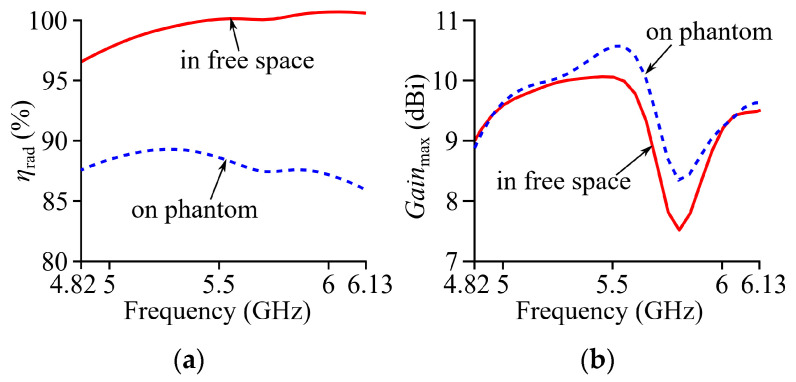
Simulated curves of (**a**) *η*_rad_ and (**b**) *Gain*_max_ with respect to frequency of Ant II in flat condition.

Figure 8 shows the simulated curves of the radiation efficiency *η*_rad_ and maximum gain *Gain*_max_ of Ant II with respect to frequency within its −10 dB impedance band. The simulated *η*_rad_ and *Gain*_max_ of the proposed antenna in free space are, respectively, above 97% and 7.6 dBi. On the phantom model, the simulated *η*_rad_ is lower than the free-space scenario because of the absorption of radiation in the phantom model, but is still above 86%. On the other hand, the simulated *Gain*_max_ of Ant II on the phantom model is higher than the free-space counterpart due to the reflected backward radiation on the phantom model.

**Figure 9:**
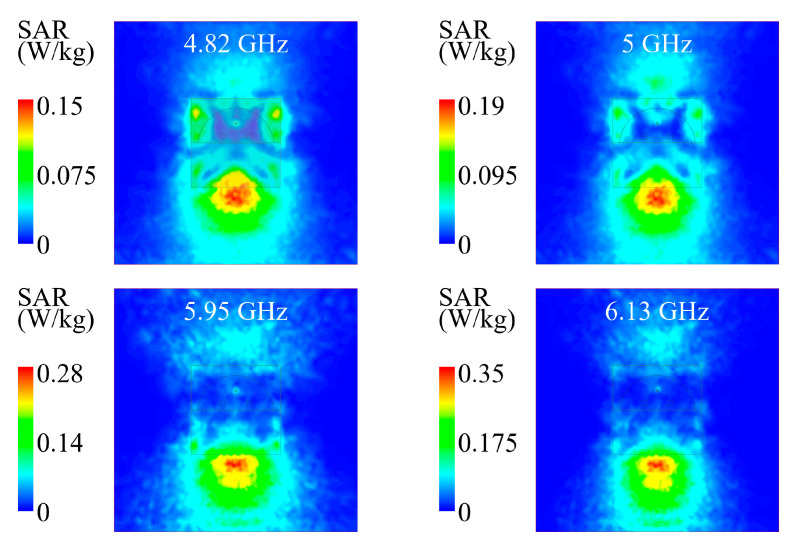
Simulated SAR distributions on the phantom model for Ant II.

The 1 g average SAR in the phantom model is simulated for Ant II with a 0.5 W input power, and obtained SAR distributions at 4.82, 5, 5.95, and 6.13 GHz are shown in Figure 9. At each frequency, the simulated maximum SARs are respectively 0.15, 0.19, 0.28, and 0.35 W/kg, which are much lower than the safety limits specified by IEEE C95.1-2005 (≤1.6 W/kg) [28] and EN 50361-2001 (≤2.0 W/kg) [29]. Therefore, it is compelling that the designed ground plane of Ant II can provide sufficient electromagnetic isolation, which effectively presents severe electromagnetic damage in the human tissue from the antenna radiation.

## 5. Prototype Fabrication and Measurement Results

**Figure 10:**
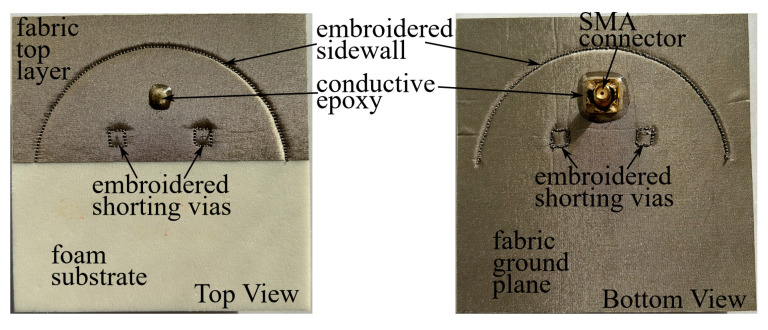
Photos of fabricated prototype of Ant II.

A textile prototype of Ant II is practically fabricated with the mentioned materials and computerized embroidery, and photos of it are shown in Figure 10. Conductive epoxy CW2460 is employed for the electrical connection and physical bonding at the feeding probe. Because of the used textile and foam materials, the fabricated antenna prototype is highly flexible.

Measurements of the |S_11_| parameter are carried out on the fabricated prototype in free space and on the body of a volunteer. The measured |S_11_| curves of Ant II are shown in Figure 6. The observed decent agreements between the measured |S_11_| curves and simulated return loss curves validate the design. In Hfss simulations, the shorting vias are modeled as ideal uniform planes with a zero thickness. In the practical fabrication, the thickness of the embroidered threads is not zero, and the effective via size *a* is therefore slightly larger than the designed value (6 mm). According to the simulations and analysis in Section 3, increasing *a* leads to a rise in *f_r_*_1_, but rarely results in a change in *f_r_*_2_ and *f_r_*_3_ because the added shorting vias do not severely change the electric field distributions in these two higher-order modes. Therefore, the slight increase in the effective value of the via size *a* due to the non-zero thread thickness can explain the observed minor difference around *f_r_*_1_ and coincidence round *f_r_*_2_ and *f_r_*_3_ between the measured and simulated |S_11_| curves. In free space, the measured −10 dB impedance band is 4.87~6.17 GHz (23.5% fraction bandwidth). The measured *f_r_*_1_ and *f_r_*_3_ are, respectively, 5.06 and 5.96 GHz, but the notch of the second mode is not deep enough to identify *f_r_*_2_. When the antenna is practically worn by the volunteer, the measured |S_11_| curve of Ant II almost overlaps with the free-space result, and the shielding effect of the ground plane is therefore well verified.

**Figure 11:**
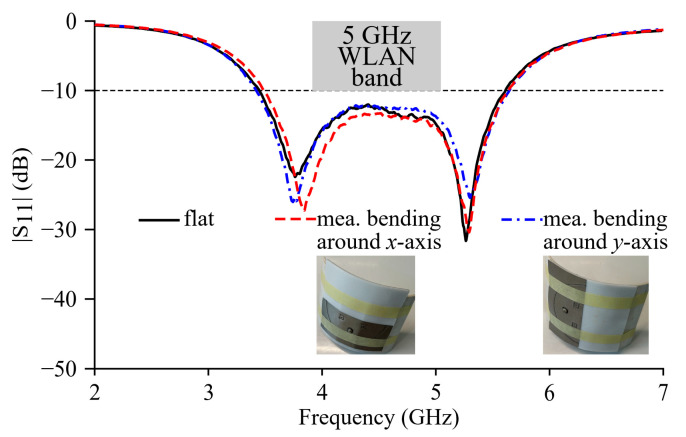
Comparison between measured free-space |S_11_| curves of Ant II in flat and cylindrical bending conditions.

Measurements are carried out on the prototype bending along a cylindrical surface with an 8 cm radius to imitate the practical situation where the antenna is located on the shoulder or limbs of the wearer, and the measured |S_11_| curves are shown in Figure 11. With bending conditions around the *x*-axis or *y*-axis, the observed shift in the resonance frequencies does not necessarily lead to a notable deterioration in the antenna’s performance. This therefore indicates the robust frequency characteristic of Ant II against physical deformations in practical wearable applications.

**Figure 12:**
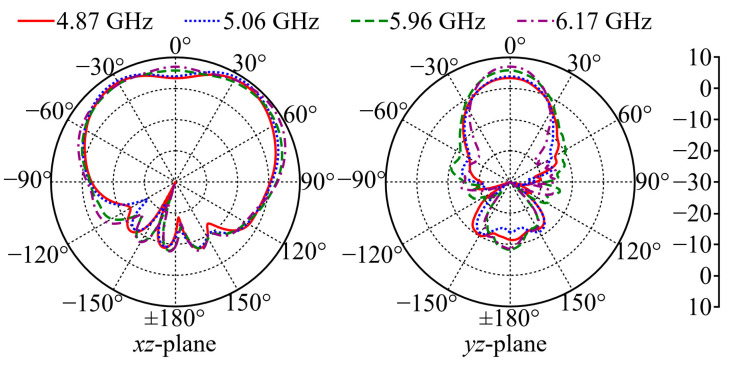
Measured coplanar-polarized gain patterns of Ant II in free space.

Figure 12 shows the measured free-space coplanar-polarized gain patterns at 4.87, 5.06, 5.96, and 6.17 GHz for Ant II. Although these measured investigated frequencies are slightly lower than the simulated results, the corresponding measured patterns show an agreement with the simulation results in Figure 7a, and the measured maximum co-polarized gains are 8.3, 9, 7.9, and 9.3 dBi at each frequency, respectively. Due to the limited equipment, *η*_rad_ and SAR are not practically measured in this work.

## 6. Conclusions

A textile bandwidth-enhanced HMSIC antenna based on embroidered shorting vias was introduced. The strategy of adding an embroidered square hollow post at the intersections of the zero-E traces of the ${TM}_{210}^{HM}$ and ${TM}_{020}^{HM}$ modes as shorting vias and the optimization of the size of the square shorting vias provide a simple and effective bandwidth-enhancing method for the textile HMSIC antenna. As a continuation of our previous work [23], this article aims to provide an improved via-based bandwidth-enhancing method for the textile HMSIC antenna. The novelty of this work mainly lies in two aspects. Firstly, compared with the strategy based on two-band merging, this work proposed the strategy of merging the bands of three modes to achieve a higher −10 dB impedance bandwidth. Secondly, compared with the simultaneous optimizations of multiple parameters including the positions and quantity of the shorting vias required in reference [23], only via size *a* needs to be optimized in this work, and the parameter optimization is significantly simplified. Table 2 shows the comparison of key performance parameters between the proposed antenna and other similar wearable/flexible bandwidth-enhanced antennas, and the proposed antenna is of the leading bandwidth and radiation efficiency compared to other candidates. Simulation and measurement results prove its robust frequency response characteristic and electromagnetic safety in the proximity of human tissues and stable performance against physical deformations. Furthermore, the identical embroidery technique in building the sidewall and shorting vias makes possible its low-cost mass production.

## Figures and Tables

**Table 1:** Relative Permittivities, Conductivities, Densities and Thicknesses of Each Layers of Phantom Model.

Layer	*ε_r_*	Conductivity (S/m)	Density (kg/m^3^)	Thickness (mm)
Skin	35.11	3.72	1100	3
Fat	4.95	0.29	910	7
Muscle	48.48	4.96	1041	50

**Table 2:** Comparison with other wearable/flexible bandwidth-enhanced HMSIC antennas and PIFAs.

Reference	Frequency (GHz)	Bandwidth (%)	*Gain*_max_ (dBi)	*η*_rad_ (%)
[17]	5.5	23.7	4.3	85
[18]	2.45	6	Not given	Not given
[19]	5.5	15.7	7.1	≥94
[20]	2.42 (OFF) 2.52 (ON)	9.1 (OFF) 8.3 (ON)	5.8 (OFF) 5.1 (ON)	95 (OFF) 97 (ON)
[23]	5.5	19.7/14.7	≥9.2/9.0	≥98/95
[24]	5.5	18	5.9	74.1
This work	5.5	23.5	≥7.6	≥97

## Data Availability

All data are included within the manuscript.
